# Development of duplex PCR for differential detection of goatpox and sheeppox viruses

**DOI:** 10.1186/s12917-017-1179-0

**Published:** 2017-08-31

**Authors:** Zhixun Zhao, Guohua Wu, Xinmin Yan, Xueliang Zhu, Jian Li, Haixia Zhu, Zhidong Zhang, Qiang Zhang

**Affiliations:** Key Laboratory of Animal virology of the Ministry of Agriculture, State Key Laboratory of Veterinary Etiological Biology, Lanzhou Veterinary Research Institute, CAAS, Lanzhou, People’s Republic of China

**Keywords:** Goatpox virus, Sheeppox virus, Duplex PCR assay, Differential diagnosis

## Abstract

**Background:**

Clinically, sheeppox and goatpox have the same symptoms and cannot be distinguished serologically. A cheaper and easy method for differential diagnosis is important in control of this disease in endemic region.

**Methods:**

A duplex PCR assay was developed for the specific differential detection of Goatpox virus (GTPV) and Sheeppox virus (SPPV), using two sets of primers based on viral E10R gene and RPO132 gene.

**Results:**

Nucleic acid electrophoresis results showed that SPPV-positive samples appear two bands, and GTPV-positive samples only one stripe. There were no cross-reactions with nucleic acids extracted from other pathogens including foot-and-mouth disease virus, Orf virus. The duplex PCR assay developed can specially detect SPPV or GTPV present in samples (*n* = 135) collected from suspected cases of Capripox.

**Conclusions:**

The duplex PCR assay developed is a specific and sensitive method for the differential diagnosis of GTPV and SPPV infection, with the potential to be standardized as a detection method for Capripox in endemic areas.

## Background

Goatpox virus (GTPV) and sheeppox virus (SPPV) are members of the genus Capripoxvirus (CaPV) of the family Poxviridae, which also contains the lumpy skin disease virus (LSDV). GTPV and SPPV can cause systemic poxviral diseases of domesticated small ruminants [1–3], which are classified as notifiable diseases by World Organization for Animal Health (OIE). Morbidity and mortality varied with different animal species [4]. Mild infections are usual among native breeds in endemic areas and the morbidity ranges from 1% to 75% or higher [5]. In contrast, more severe clinical symptoms are observed in animals with concurrent infections, stressed or young animals, or animals from regions where the poxviral diseases have not happened for long time. The mortality was close to 100% in these highly susceptible animals [6]. The genome of CaPV is about 150 kbp of double-stranded DNA, which shares more than 147 putative genes, including conserved poxvirus structural and replicative genes, and genes likely involved in host range and virulence [7]. Sheeppox and goatpox is an economically important disease in goat and sheep-producing areas of the world [8]. Sheeppox and goatpox have the same symptoms and are clinically distinguishable. For accurately and promptly controlling any outbreak of sheeppox and goatpox, the foremost requirement is a specific tool for differential detection of the causative agents. Digestion of CaPV p32 gene with Hinf I and sequence alignment of GpCR gene were developed to discriminate GTPV and SPPV [9, 10]. In this study, a duplex polymerase chain reaction (PCR) assay based on the E10R and RPO132 gene was developed to distinguish GTPV and SPPV. The performance of the assay was evaluated with clinical samples. In comparison with the PCR-RFLP, the newly established duplex PCR assay is a time-efficient and simple alternative for differential diagnosis of GTP and SPP that could be used as a diagnostic tool in clinical samples.

## Methods

### Gene sequences and primers design

**Fig. 1:**
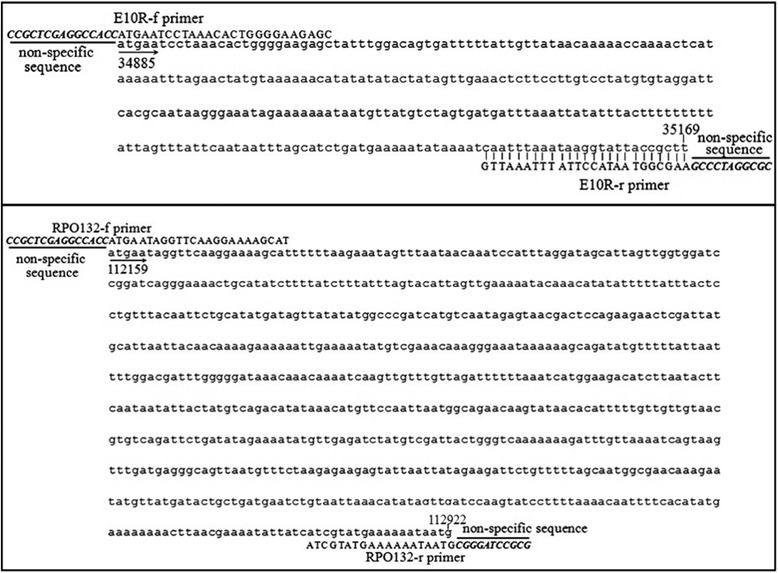
Target gene sequences and primers. Nucleotide sequences of the E10R and RPO132 amplicon (E10R, RPO132 GenBank accession no. AY077832.1) and locations of the primers along the sequence

PCR primers were designed using Primer Premier 5.0 software based on E10R gene and RPO132 gene of GTPV and SPPV E10R gene. Selection of these targets for primer designs were based upon previous bioinformatics analyses of CaPV genomes and corresponding homologs from other near-neighbor viruses listed in the NCBI (National Center for Biotechnology Information) database (data not shown). Nucleotide sequences of the primers are shown in Table 1 and Fig. 1. A nonspecific sequence highlighted in bold was added at 5′ end of each primer (E10R-f primer: “CCGCTCGAGGCCACC”; E10R-r primer: “CGCGGATCCCGC” RPO132-f: “*CGCGGATCCCGC*” and RPO132-r: “*CCGCTCGAGGC CACC*”).

**Table 1:** Primer designed to detect goatpox and sheeppox virus by duplex PCR

Primer name	Length	Sequence(5′-3′)	Notes
E10R-f	41	*CCGCTCGAGGCCACC*ATGAATCCTAAACACTGGGGAAGAGC	The universal primers for GTPV and SPPV RPO40 gene, the predicted length of PCR is 285 bp.
E10R-r	36	*CGCGGATCCCGAAGCGGT*AATACCTTATTTAAATTG	
RPO132-f	39	*CCGCTCGAGGCCACC*ATGAATAGGTTCAAGGAAAAGCAT	The special PCR primers for SPPV RPO132 gene, the predicted length of Lamp is 746 bp.
RPO132-r	30	*CGCGGATCCCGC*ATTATTTTTTCATACGAT

### Viruses

SPPV Gulang/2009 strain was isolated and adapted in Vero cell culture. GTPV AV40 vaccine strains adapted in Vero cell culture was obtained from the Institute of Veterinary Drug Control, Beijing, China. The nucleic acid extracted from foot-and-mouth disease virus (FMDV) isolates was provided by the National Foot-and-Mouth Disease Reference Laboratory, LVRI, and the nucleic acids extracted from *Mycoplasma ovipneumoniae* (*M. ovipneumoniae*)*, Chlamydia psittaci, L. interrogans, Toxoplasma gondii* and *Babesia* were provided by the Key Laboratory of Veterinary Parasitology of Gansu Province, LVRI. The clinical samples collected from suspected cases of goatpox and sheeppox were stored in PBS, pH 7.4 and a 10% (*w*/*v*) suspension was made for extraction of total gDNA using commercial DNA extraction kit (TaKaRa, Dalian, China) according to the manufacture’s instruction.

### Reaction mixtures and optimal duplex PCR conditions

Duplex PCR reactions were carried out in a volume of 25 μL containing 10 μL premix Taq (TaKaRa, Dalian, China), 0.2 μM of each of the E10R-f and E10R-r primers, 1.0 μM of each of the RPO132-f and RPO132-r primers and 2 μL (100 ng) of extracted SPPV or GTPV gDNA as template. The duplex PCR assay was performed at the following conditions: initial denaturizing at 94 °C for 4 min, followed by 34 cycles of 94 °C for 30 s, 50 °C for 30 s, 72 °C for 45 s and final extension of 72 °C for 5 min.

### Analysis of duplex PCR products

Products were separated electrophoretically in 1% agarose gel (Gelrose TM, Life Technologies, USA) containing 0.5 g/ml ethidium bromide, followed by visualization under ultraviolet (UV) light. 6 μL of duplex PCR products was used for visualization and the electrophoresis was done for 20 min at a constant 120 V. After visualization, the picture was documented using a gel documentation system (Peiqing Image Biosystem, Shanghai).

### Duplex PCR sensitivity

The sensitivity of the duplex PCR assay was tested using 10-fold serially diluted GTPV (1.011 × 10^10^–1.011 × 10^0^ copies per μL) or SPPV DNA (1.043 × 10^10^–1.043 × 10^0^ copies per μL) as template.

### Duplex PCR specificity

The specificity of the duplex PCR assay was evaluated by using purified the nucleic acids extracted from sheep, goats and some wild ruminants, SPPV, GTPV, Orf, FMDV, *M.ovippneumoniae, Chlamydia psittaci, L.interrogans, Toxoplasma gondii, Babesia* and a control without template was also included as a negative control in each test. The duplex PCR products were sequenced to further confirm the specificity of the assay.

### Evaluation of the duplex PCR assay in clinical samples

The performance of the duplex PCR assay was evaluated with 135 clinical samples preserved in our laboratory. These samples were pre-screened by PCR-RFLP [9, 10] and LAMP [11] as previously reported. Among them 48 samples were collected from goats infected with GTPV and 87 from sheep infected with SPPV. Animal experimentations were performed inside the biosafety facilities of the Lanzhou Veterinary Research Institute, Chinese Academy of Agricultural Sciences (LVRI, CAAS), in compliance with the regulations of the Animal Ethics Procedures and Guidelines of the People’s Republic of China (AEPGPRC).

## Results

### Primers and gene sequences

Several GTPV and SPPV genomic sequences were downloaded from GenBank and aligned with each other using the MegAlign. The most conserved segments within the E10R and RPO132 genes of GTPV and SPPV were selected as the targets. All primers were designed by the software Primer Premier 5.0. Two pairs of primers were used for the duplex PCR assay, i.e., E10R primers (E10R-f and E10R-r) and RPO132 primers (RPO132-f and RPO132-r). The sequences of all primers were shown in Table 1.

### Optimization of duplex PCR reaction conditions

**Fig. 2:**
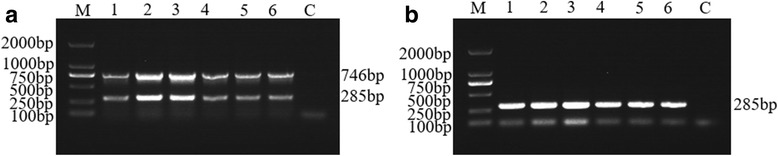
Optimization of annealing temperature (Tm) for duplex PCR reaction in the detection of GTPV or SPPV using mix primers. Agarose gel electrophoresis showing the effect of Tm on duplex PCR. **a** Duplex PCR amplicated products using 100 ng SPPV gDNA as template. **b** Duplex PCR amplicated products using 100 ng GTPV gDNA as template. Lane 1–6 is 46 °C, 48 °C, 50 °C, 52 °C, 54 °C and 56 °C, respectively; Lane M: 2000 bp DNA Ladder Marker (TaKaRa, Dalian) and Lane C: No template control

The SPPV specific primers (RPO132) amplified approx. 746 bp fragments and the GTPV specific primers (E10R) amplified approx. 285 bp fragments as expected, when visualized in 1% agarose gel using ethidium bromide staining (Fig. 2).

**Fig. 3:**
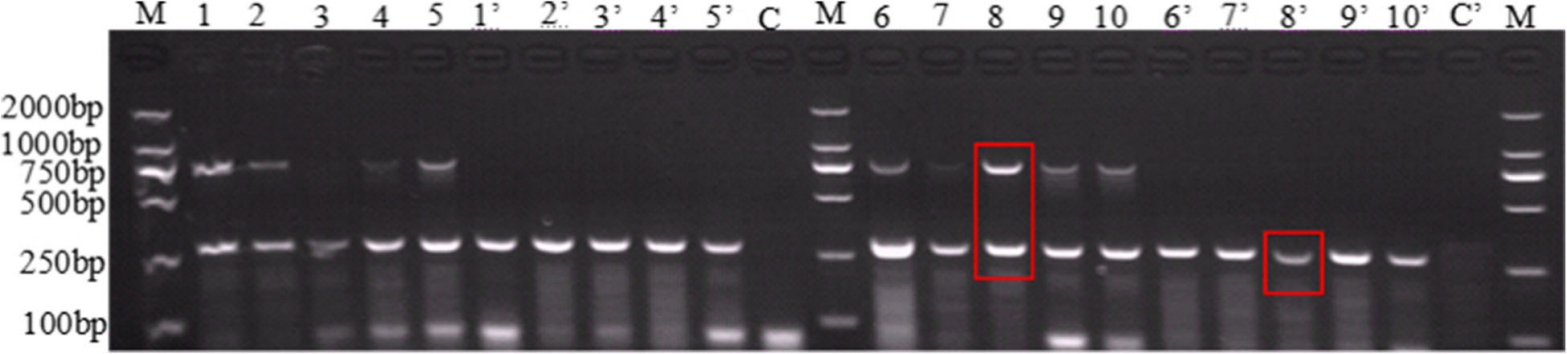
Optimization of primers rate for duplex PCR reaction. Lane 1–5 and 1′-5′: E10R primers is 0.5 μM, RPO132 primers is 0.2 μM, 0.4 μM, 0.6 μM, 0.8 μM and 1 μM, respectively; Lane 6–10 and 6′-10′: RPO132 primers is 0.5 μM, E10R primers is 0.2 μM, 0.4 μM, 0.6 μM, 0.8 μM and 1 μM, respectively. Lane1–5 and 6–10: 100 ng SPPV genome as templates; Lane1’-5’and 6′-10′: 100 ng GTPV genome as templates, respectively. Lane C and C′: No template control. Lane M:2000 bp DNA Ladder Marker (TaKaRa, Dalian). The strip in red grid showed the result is better using 1 μM RPO132 primers and 0.2 μM E10R primers in the reaction

To determine the optimal temperature for amplification in the duplex PCR assay, a range of temperatures from 46 °C to 56 °C were tested. The results showed that the targeted GTPV and SPPV genes can be amplified at all of above annealing temperature using E10R and RPO132 mix primers, respectively. As shown in Fig. 2 the strongest amplified products were observed at 50 °C. In the optimization of ratio of E10R to RPO132 primers, it was found that

the targeted genes can be amplified at any proportion of E10R and RPO132 primers, respectively. However, the best amplification occurred when each of RPO132 primers is 1 μM and each of E10R primers is 0.2 μM, respectively (Fig. 3).

**Fig. 4:**
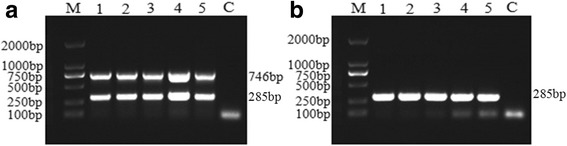
Optimization of reaction cycles for duplex PCR reaction in the detection of GTPV or SPPV using mix primers. Agarose gel electrophoresis showing the effect of cycles on duplex PCR. **a** Duplex PCR amplicated products using 100 ng SPPV gDNA as template. **b** Duplex PCR amplicated products using 100 ng GTPV gDNA as template. Lane 1–5: Reaction annealing temperature is 50 °C for 20 cycles, 25 cycles, 30 cycles, 35 cycles and 40 cycles; Lane C: No template control and Lane M: 2000 bp DNA Ladder Marker (TaKaRa, Dalian)

In determination of the best reaction cycles, the results showed that the targeted genes can be produced after 25 cycles to 40 cycles using the optimized concentration of the primers at 50 °C, and the amplified products at 35 cycles was clearer (Fig. 4) than other cycle conditions.

### Duplex PCR sensitivity for detection of GTPV and SPPV

**Fig. 5:**
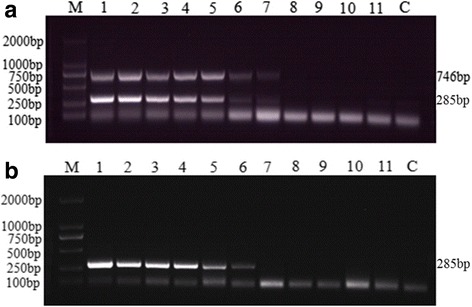
Duplex PCR sensitivity. Amplification using different concentration gradient of the gene as template, nucleic acid electrophoresis test results. **a** Lane 1–10: SPPV gDNA concentration gradient for 1.011 × 10^10^–1.011 × 10^0^ copies as template, (**b**) Lane 1–10: GTPV gDNA concentration gradient for 1.043 × 10^10^–1.043 × 10^0^ copies as template, respectively. Lane M: 2000 bp DNA Ladder Marker (TaKaRa, Dalian) and Lane C: No template control

To determine the sensitivity of the duplex PCR assay developed, a serial dilutions of the purified genome DNA (gDNA) of SPPV and GTPV were used. The concentration of the purified viral gDNA was measured by NanoDrop 2000 (Thermo Scientific). SPPV Gulang/2009 gDNA was ten-fold diluted ranging from 1.011 × 10^10^–1.011 × 10^0^ copies and GTPV AV40 gDNA was diluted ranging from 1.043 × 10^10^–1.043 × 10^0^ copies respectively. After amplification under the optimized conditions as described above 6 μL PCR amplified products e was tested by nucleic acid electrophoresis, and then was observed by UV gel imaging system. The results showed that the duplex PCR assay was able to specifically amplify the SPPV gDNA from 1.011 × 10^10^–1.011 × 10^4^ copies, and the control group had no stripe (Fig. 5a). 1.043 × 10^5^ copies of GTPV gDNA template was detected (Fig. 5b, Table 2).

**Table 2:** Comparison of LAMP method [19] sensitivity with the duplex PCR

Methods	Templates	Sensitivity (copies/reaction)									
		10^9^	10^8^	10^7^	10^6^	10^5^	10^4^	10^3^	10^2^	10^1^	10^0^
Duplex PCR	GTPV	+	+	+	+	+	−	−	−	−	−
Duplex PCR	SPPV	+	+	+	+	+	+	−	−	−	−
GTPV LAMP	GTPV	+	+	+	+	−	−	−	−	−	−
SPPV LAMP	SPPV	+	+	+	+	+	+	−	−	−	−
GSPV LAMP	GTPV/SPPV	+	+	+	+	+	+	+	−	−	−

Note: Detection of about 100 ng GTPV AV40 or SPPV Gulang/2009 genome DNA using different methods. “+” stand for positive result and “-” stand for negative result

### Duplex PCR assay specificity

**Fig. 6:**
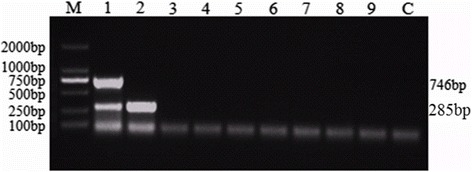
Specificity of duplex PCR for detection different pathogen nucleic acid. Aboat 100 ng DNA or cDNA template of ten different sheep or goat pathogens were used in LAMP reaction. Agarose gel electrophoresis (1%) of PCR products stained with Ethidium bromide and visualized under UV transilluminator. Lane 1: SPPV; Lane 2: GTPV; Lane 3: *Orf* virus; Lane 4: FMDV O/China 99; Lane 5: *M. ovippneumoniae*; Lane 6: *Chlamydia psittaci*; Lane 7: *L.interrogans*; Lane 8: *Toxoplasma gondii*; Lane 9: *Babesia*; C: No template control and Lane M: 2000 bp DNA Ladder Marker (TaKaRa, Dalian)

In investigation of the specificity of the duplex PCR assay, the reactions were performed using a panel of genomes extracted from GTPV, SPPV, Orf virus, FMDV, *M. ovippneumoniae*, *Chlamydia psittaci*, *L.interrogans*, *Toxoplasma gondii* were tested, respectively. The duplex PCR assay was shown to be specific for the detection of GTPV and SPPV, respectively and there was no cross-reaction with genome of Orf, FMDV, *M. ovippneumoniae*, *Chlamydia psittaci*, *L.interrogans*, *Toxoplasma gondii*, *Babesia*, and without template control (Fig. 6). The duplex PCR products were then sequenced and found to match with the corresponding nucleotide sequences of E10R or RPO132 gene (data not shown). Phylogenetic tree analysis showed that the SPPV Gulang/2009 and other isolated SPPVs were distinctly different from GTPVs and LSDVs.

### Performance of the duplex PCR assay on clinical samples

All the clinical samples (*n* = 135) were detected by the duplex PCR assay, and the results were then compared to the PCR-RFLP assay [9, 10] and LAMP method [11]. The results showed that 48 samples were determined to be positive for GTPV and 87 samples were positive for SPPV by the duplex PCR assay, respectively, which was 100% consistent with the LAMP method and PCR-RFLP assay (Table 3).

**Table 3:** Results of different methods detection with clinic samples

Methods	Sample size	Positive result	Negative result	Positive detection rates (%)	Sample category
PCR-RFLP [9, 10]	135	135	0	100	48 GTPV samples and 87 SPPV samples
GSPV lamp [19]	135	135	0	100	
GTPV lamp [19]	135	48	87	100	
SPPV lamp [19]	135	86	49	98.8	
Duplex PCR	135	135	0	100	

## Discussion

GTPV and SPPV genomes are approximately 150kbp double-stranded DNA, which share at least 147 putative genes, including conserved poxvirus replication and structural genes and genes likely involved in virulence and host range [7]. Restriction endonuclease analysis and cross-hybridization studies of SPPV and GTPV indicate that these viruses, although closely related (estimated 96 to 97% nucleotide identity), can be distinguished from one another and may undergo recombination in nature. Several PCR tests have been developed for the detection of CaPV [12–19], but only PCR-RFLP assay was developed to distinguish them in the beginning. However, PCR-RFLP assay is time consuming, and expensive and requires a high degree of laboratory experience in molecular biology. Then a LAMP method was developed for the specific differential detection of GTPV and SPPV, using three sets of LAMP primers designed on the basis of the ITRs [11]. LAMP for distinguishing GTPV and SPPV was performed at 62 °C in 45–60 min. However, the operation of the experiment requires extreme caution because of its high sensitivity leading to contamination. There is a real need for a more convenient alternative to PCR that is inexpensive, and easy to operate and maintain.

**Fig. 7:**
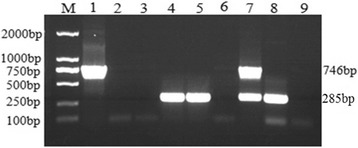
Single PCR and duplex PCR for detection different GTPV and SPPV nucleic acid using E10R primers and RPO132 primers or mix primers. Lane 1–3: single PCR detect 100 ng SPPV gDNA, 100 ng GTPV gDNA and no template using RPO132 primers; Lane 4–6: single PCR detect 100 ng SPPV gDNA, 100 ng GTPV gDNA and no template using E10R primers; Lane 7–9: duplex PCR detect 100 ng SPPV gDNA, 100 ng GTPV gDNA and no template using mixture of RPO132 primers and E10R primers. Lane M: 2000 bp DNA Ladder Marker (TaKaRa, Dalian)

E10R is encoded by ORF40 gene and RNA polymerase subunit RPO132 is encoded by ORF116 gene. The E10R sequence of GTPV and SPPV showed high homologies to VACV E10R sequence, and RPO132 sequences of GTPV and SPPV showed high homologies to VACV A32R sequence. Initially, we found E10R primer designed based on SPPV E10R gene can amplify target gene from SPPV genome, also can amplify target gene from GTPV genome. RPO132 primer designed based on SPPV RPO132 gene only can amplify target gene from SPPV genome, but cannot amplify target gene from GTPV genome. But both annealing temperature of E10R and RPO132 primers will not be able to adjust to a consistent system. We initially planned to express E10R and RPO132, and inserted them into expression vector by enzyme restriction sites we added in the primers. So, non-specific sequences including Kozak sequence, restriction enzyme sites and protective bases or only restriction enzyme sites and protective bases sequences were added located in front of the initiation codon in upstream primer or added located behind termination codon in downstream primer, respectively. Accidentally, we found the above primers added non-specificity sequence can amplify the specificity target and the annealing temperature can be consistent at the same time in one system. E10R and RPO132 primers adding the nonspecific sequence can differential detect SPPV and GTPV obviously (Fig. 7). That is to say, added nonspecific sequence E10R and RPO132 primers mixture can specific amplification two target genes sizes obvious differences from SPPV genome, respectively, and can only amplify one gene from GTPV genome in this study (Fig. 7). Related experiments showed that the sequences of the duplex PCR primers can specificity amplify two target genes from SPPV nucleic acid, but only one target gene can be amplified from GTPV nucleic acid under the same conditions. Further experiments proved that this duplex PCR analysis can fully distinguish between GTPV and SPPV.

One hundred thirty-five epidemic materials preserved in our laboratory were tested using duplex PCR diagnosis method, the results were consistent with the laboratory tested results (Table 3), which suggests the duplex PCR is able to detect the clinal samples.

## Conclusion

The present study showed that the duplex PCR method of differential detection of GTPV and SPPV is highly specific, and sensitive. Thus, it might be the optimal detection system for field detection and differential diagnosis of GTP and SPP. It is a promising assay for extensive application for the diagnosis of GTPV and SPPV infection in the laboratory and field, especially in countries that lack the resources needed for molecular diagnostic techniques.

## Abbreviations

CaPV: Capripoxvirus
gDNA: Genome DNA
GTPV: Goatpox virus
LSDV: lumpy skin disease virus
ORF: Open reading frame
PCR-RFLP; RFLP-PCR: Polymerase chain reaction-restriction fragment length polymorphism
SPPV: Sheeppox virus
UV: Ultraviolet light
